# Improving motivation among primary health care workers in Tanzania: a health worker perspective

**DOI:** 10.1186/1478-4491-4-6

**Published:** 2006-03-07

**Authors:** Rachel N Manongi, Tanya C Marchant, Ib Christian Bygbjerg

**Affiliations:** 1 Department of Community Health, Tumaini University, Kilimanjaro Christian Medical College, Moshi, Tanzania; 2 Department of Public Health and Policy, London School of Hygiene and Tropical Medicine, London, UK; 3 Department of International Health, Institute of Public Health, University of Copenhagen, Denmark

## Abstract

In Tanzania access to urban and rural primary health care is relatively widespread, yet there is evidence of considerable bypassing of services; questions have been raised about how to improve functionality.

The aim of this study was to explore the experiences of health workers working in the primary health care facilities in Kilimanjaro Region, Tanzania, in terms of their motivation to work, satisfaction and frustration, and to identify areas for sustainable improvement to the services they provide.

The primary issues arising pertain to complexities of multitasking in an environment of staff shortages, a desire for more structured and supportive supervision from managers, and improved transparency in career development opportunities. Further, suggestions were made for inter-facility exchanges, particularly on commonly referred cases.

The discussion highlights the context of some of the problems identified in the results and suggests that some of the preferences presented by the health workers be discussed at policy level with a view to adding value to most services with minimum additional resources.

## Introduction

In 1992 the Tanzanian Ministry of Health (MoH) reviewed the national primary health care (PHC) strategy and decentralized primary health care delivery from national level to district level. It was envisaged that making local governments responsible for staffing and maintaining health centre and dispensary-based facilities would improve the provision of services [1]. The strategy also incorporated continuing education to health workers as a means of improving their knowledge and skills and as an important motivation factor.

The MoH has developed career development structures for each category of health worker, including criteria for upgrading. Other incentives for health workers may include housing and appreciation of good performance as well as improvement of communication between different levels of the health system [2]. Guidelines for effective and efficient health care delivery require District Health Management Team (DHMT) members to directly supervise each PHC facility at least once a quarter [3].

Despite relatively widely distributed urban and rural health care services in Tanzania, questions about the functionality of the primary health care facility (PHCF) infrastructure still remain. Several Tanzanian studies have explored user satisfaction with health services and quality of care given to users and found weaknesses in both structural and process quality aspects of care given to users [4-7]. The quality of health services, their efficacy, efficiency, accessibility and viability depend mainly on the performance of those who deliver them [8]. From the literature, performance has been associated with training policies and improving health workers' availability and retention [9,10]. However, in Tanzania only one study has investigated job satisfaction: among rural medical aides who were providing oral health care [11].

The aim of this study was to explore the experiences of primary health care workers in northern Tanzania. Their perspective of the services they provide, what motivates, satisfies and frustrates is presented, together with health care worker recommendations for how best to restructure these facilities realistically.

## Methods

### Ethical considerations

Clearance to carry out this study was obtained from the National Institute of Medical Research (NIMR) as part of a Joint Malaria Programme. The Tumaini University Research and Ethics Committee also approved the study. The study details were also discussed with relevant members of DHMTs in all the districts. Before starting the focus-group discussions, written individual consent to participate was sought after the study's aim, methods and benefits were explained. The researchers also explained that refusal to participate would have no negative implications for staff.

### Study area and population

The study was conducted from May to July 2004 in three districts: Moshi Urban, Moshi Rural and Hai districts in the Kilimanjaro region, northern Tanzania

Multiple cadres of health staff working in government primary health care facilities, from nurse auxiliary to assistant medical officer, were invited to contribute to the investigation.

Six focus-group discussions (FGDs) were conducted in the three districts, two in each district, one with nurses and one with clinicians, each consisting of 8–12 participants.

Specific information raised by health workers on promotion, training and supervision was investigated further in in-depth interviewers with the relevant District Medical Officer (DMO) with a view to confirming factual issues and to discussing constraints from a management perspective. These were tape-recorded and field notes were taken.

### Sampling

From each district a list of all health workers employed in government dispensaries and health centres was obtained. The list was further stratified to nurses (all cadres) and clinicians (all those qualified to prescribe drugs to patients). Using a random table method, 12 nurses and 12 clinicians in each district were sampled and invited to participate in a FGD. Initial contact concentrated on explaining sampling, objectives of the study and the voluntary nature of participation. A list of all cadres of staff is shown in Table 1. The government health facilities were chosen primarily because the government health system is the backbone of the Tanzanian health system and employs most health workers, compared to the private sector.

**Table 1:** Qualifications of the cadres who participated in FGDs

**Cadre**	**Award**	**Years of training**	**Qualifications for enrolment**	**Number participating in FGDs overall**
Clinical Officer (CO) (Medical Assistant)	Diploma	3	Pre-service for form IV and VI leavers	8
Clinical Officer (CO) (Medical Assistant)	Diploma	5	In-service for Rural Medical Aide – who upgraded.	8
*Rural Medical Aides (Assistant Clinical Officer)	Certificate	2	In-service – on-the-job training	16
Mother and Child Health Aides (MCHA)	Certificate	2	Pre-service course for form IV	7
Trained nurse midwife, grade B	Midwife certificate	4	In-service	7
General nurse, grade A	Nursing diploma	4	Pre-service for form IV	2
Medical attendant (Nurse auxiliary)	Certificate	1	In-service for STD VII & form IV leavers	16

Source: Ministry of Health (1995)

* The government is no longer training Rural Medical Aides. For those working with PHCFs, the term used is Assistant Clinical Officer.

### Data collection

As a preliminary investigation a pilot study was carried out in a rural district outside the main study area. Four FGDs were conducted, two with nurses and two with clinicians. The findings were used to develop an interview guide.

A semi-structured interview guide was used in the main study, which allowed flexibility within the discussion. This also allowed for additional questions on emerging themes to be incorporated using a continuous validation process.

The FGDs were conducted in Kiswahili by an experienced facilitator, assisted by the first author, who was the principal investigator (PI). An experienced nurse researcher (recorder) from the Community Health Department at Kilimanjaro Christian Medical Center (KCMC) took field notes. At the same time all the FGDs were tape-recorded and subsequently transcribed for thematic analysis by the PI (who is fluent in English and Swahili, so no intermediate interpreter was needed). Each session lasted from one hour to one hour and 30 minutes.

Each of the six FGDs was opened with a broad study question from the facilitator. Participants were asked to think about the functionality of the primary health care service and any issues that they felt inhibited their performance: participants seemed eager to be given the opportunity to discuss the topic. After the FGD had begun, the facilitator asked questions only when a priority area of the topic guide was not being addressed or when clarification was needed. Comments were occasionally given to move the talk to another level or to clarify issues.

### Analysis

A thematic analysis [12,13] was performed on the transcripts through:

• familiarization with the data, reading of the field notes and transcribing of the data. Initial coding was developed from issues emerging from the familiarization stage.

• the process of applying codes to the data. Textual codes were used to identify specific pieces of corresponding data and differing themes.

• development of themes emerging from the discourse.

## Results

From the three districts 64 participants overall attended the six FGDs. In each district the whole range of cadres was represented (from nurse auxiliary to Clinical Officer). Both male and female professionals were included, with an age range of 25 to 59 years.

In all groups the participants were keen to discuss the issue of motivation and satisfaction among health workers. Among the themes that emerged from the FGDs, three were of particular relevance to this study and were voiced across all the FGDs: division of labour; training, supervision and feedback; and promotion. A summary of results is presented in Table 2.

**Table 2:** Summary of results

**Problem**	**Proposed solutions by health workers**	**Recognized by DMO**	**Major constraints expressed by DMO**
*Training*	1. Give untrained health workers formal training to acquire skills needed	Yes	1. Not enough funds allocated
	2. In-service training	Yes	2. Understaffing
*Understaffing*	Nurses currently working as administrators should be replaced by non-medical staff	Yes	Not enough funds to employ new staff.
*Acting upwards*	None	Yes	None
*Acting downwards*	None	No	
*Gambling with health*	Equip all health facilities with microscopes	Yes	Guidelines for personnel in dispensaries doesn't include laboratory staff.
			Not enough laboratory technician/assistants trained
*Referral and feedback*	1. Specialist should visit health facilities regularly to conduct site training on cases the health workers normally refer to them	Yes	None
	2. Completed referral letter should be returned to referring clinic	Yes	None
*Supervision*	1. Feedback to be given on previous supervision visits	Yes	1. Time constraints due to intense workload of DMOs. Also, DMO activities (and resources) still partially controlled by central government.
	2. Recognition from DMO of hard work they are doing	Yes	2. None
*Promotion*	Timely promotions as outlined by Ministry guidelines	Yes	Have no final decisions; can only recommend to higher authorities

### Division of labour

Respondents in all the FGDs raised the issue that understaffing of key personnel within the PHC facilities resulted in various negative consequences for service provision – some shared across cadres and some unique to different positions.

#### Understaffing

The understaffing issue was summarized succinctly by a female nurse auxiliary who described it this way: "...Say at every centre you have got one nurse and one doctor. If it happens that the doctor faces a problem the nurse will be alone. Now she will do the cleaning and dispense drugs and deal with patients.... You often find that work to be done by two or three different people is performed by a single person".

#### Acting upwards

Many health workers felt they were forced to handle cases for which they were not trained. A nurse auxiliary was doing the work of a pharmacist and a clinician: dispensing drugs, giving injections, dressing wounds and assisting in the labour ward.

One female nurse auxiliary had this to say "....myself I'm handling these patients more compared to those of higher cadres...we should be given the opportunity to go for training so as to handle our patients properly..."

#### Acting downwards

When lower cadres were acting upwards in service provision, the result was often that non-medical work also had to be shared. Here there was evidence of an urban/rural divide, with rural clinical staff, but not urban, accepting the necessity of sharing all responsibilities – including cleaning – when lower cadres were acting upwards. An urban (female) clinical officer complained: "I am not happy to do cleanliness when I am already overworked without getting anything. No, I can't do cleaning."

In contrast, a rural (male) assistant clinical officer reported: "Though we have much work to do, cleanliness is important. Without cleanliness your medical profession will be meaningless. So we (the clinicians) clean up around us to care for the patient's health and also our own health."

#### Gambling with the health of patients

Across all FGDs health workers were aware that some community members looked for quality they simply could not produce. The lack of laboratory facilities alone forced them to treat patients by trial and error, which was compared with gambling.

One female assistant clinical officer said: "We don't have a microscope or even a laboratory. So we are only doing diagnosis and using our experience to decide. This is like playing a game of chance (Kamari) for the money as you are not sure if you are treating malaria or typhoid or both. I do feel hurt more than the patient himself. This is really discouraging for us working in these dispensaries."

The same woman went on to say: "People in the community nowadays know what quality of services they want. They usually ask 'Why don't I go to the hospital where there are medical officers and working facilities instead of going to the dispensaries where there is no laboratory and facilities?' I think the community should be allowed to go where there are good services."

#### Training, supervision and feedback

Given the environment already described – of a shortage of clinical staff in situ and more junior cadres acting upwards on a regular basis, this section highlights a number of issues related to training, the use of skills of health workers at all levels and the frustrations experienced in career development.

#### Referrals and feedback

Across all three districts the clinical officers felt they often saw cases (especially obstetric/gynaecological conditions, sexually transmitted illnesses, skin disease) that they could have handled better given some specific training. For the patient, there was a considerable cost attached to being referred to a district hospital and often relatives were unwilling or unable to pay for the transfer and subsequent care implied.

This was felt to be particularly true of obstetric cases when women without adequate financial resources commonly wait until the last possible moment to seek care and then may not be able to travel and gain access to emergency services. Understandably these cases deeply affected the health workers' morale. One male assistant clinical officer said: "One day in a health centre where I was working, a mother came around with labour pains. I was called from home to try to help. She managed to deliver normally but she had a postpartum haemorrhage. I did vaginal packing and elevated the bed while looking for any means of transport possible to a district hospital. We didn't get the transport until 6 a.m. [and] by that time it was too late. Everything was finished."

Further, if a patient was referred, considerable frustration was expressed regarding the lack of subsequent feedback. A female assistant medical officer said: "There are cases we refer just because we don't know how to go about them and we don't get feedback; next time when we get a similar case our job is just to refer. We don't learn anything."

#### Experienced but not trained

For less-qualified cadres the situation was seen quite simply. Due to the circumstances of their colleagues it was necessary from time to time to deal with things that they had no training to handle. A commonly heard quote from the FGDs was "We are not trained; it is just the working experience we use to manage".

This was raised in the in-depth interviews with the DMOs, who recognized the problem but said that as managers they were financially constrained. All three DMOs explained that under the former system the government examined all applicants for further training and sponsored successful applicants. However this has changed: "Nowadays the government has pulled out from sponsoring these health workers. Every district is supposed to upgrade the health workers according to their needs. The problem is money. We don't have an allocation for that. In my district I have told the health workers to apply for schools they want. Once they get admission, we pay half of the fees and they have to pay the rest".

Health workers in all the FGDs proposed a number of coping strategies. More sharing of information with other medical professionals was requested. They felt that it was time for them to be visited by specialists for training purposes "...These doctors who are specialists or those who are above our level should visit us once in awhile to discuss with us the management of those cases which we normally refer to them" (female clinical officer).

Improving systems for feedback to clinics from referral hospitals on case management was highlighted as another practical way of learning without creating a financial burden to the health service.

Many participants wanted to see a rotation system implemented within Regions and between staff of the same qualification as a means of keeping up and learning new skills: "...It will be a good idea if we do rotations. After two years we go back to work in a district /regional hospital and another group from there comes to take our places....after all we all have the same qualifications" (male clinical officer). Another clinical officer added "It will be good and motivating if we meet and share experience among ourselves. ...We don't want to be paid for this; we want the DMO to organize this and to give us permission."

#### Supervision and feedback

Across the FGDs it was agreed that during supervision more negative comments were received than positive. The health workers reported that there was little or no on-site supervision from their immediate superiors and the external supervision from DHMT was irregular and not supportive. One male assistant clinical officer said: "....The supervision is not friendly and lovingly done. There should have been a plan that every three months there will be supervision but instead, after six months you can see two people having papers and pen in their hands coming asking questions like a policeman; it is not a friendly one but faults-finding supervision."

Many complained that they never received any written or oral feedback from supervisory visits. One female assistant clinical officer said:"When the re-supervision is done for the second time, there should be feedback from the first supervision so that we can recognize where we went wrong and correct our mistakes. There should also be feedback of the problems identified from the previous supervision. It is not easy to think the supervisors are useful when reported problems remain and no feedback is given."

As part of the in-depth interviews with DMOs the fulfilment of MoH recommendations for monthly supervision was raised. One DMO said: "The problem of poor supervision is not human resource or transport or allowances but poor planning centrally (MoH). Most of the time we get unplanned visitors from MoH with emergency issues to tackle with short deadlines. They say we are decentralized but in fact we are still getting orders from above, "top down". As if this is not enough, we have to organize or attend several seminars and different workshops. Almost always we are called for ad hoc meetings. These activities also require the only vehicle we have. This of course is also a problem to the rest of team members. They are facing same problems. We have to use one vehicle for the team with different activities so, sometimes other activities have to suffer."

#### Promotion

All health workers across all the FGDs knew of their right to be promoted every three years and, according to MoH regulations, that every promotion was to be accompanied by a salary increment. However, the majority of participants said they had worked for more than 10 years without being promoted, and this was mentioned as an important factor for dissatisfaction:

One female nurse auxiliary explained her situation: "You may work for seven to eight years without been given any promotion, at the same time you earn less compared to those auxiliary nurses who were employed later. Their salary scales are twice ours and we have been in work for 30 years. They tease us saying, "What are you proud of? After all we have more salary than yours."

Many respondents found promotion issues discouraging but clearly the coping strategy explained by many of the respondents was, "We are working for God". This was elaborated on by one male assistant clinical officer, who said: "You may work for 10 years and be promoted once. If your salary scale is not corrected it will continue going that way. It's really discouraging to work, but due to it being a community service, you keep on working as God will pay you in heaven one day."

A clear recommendation for alleviating the problem of distrust and dissatisfaction regarding promotion was to improve communication at two levels: first, for supervisors to clearly communicate performance appraisal to staff, and second, for improved transparency over the process of applications for promotion.

The DMOs could all clearly explain the correct procedure for promotion – that their role was to recommend to the local government the names of health workers who were due for promotion (for those with two years of training or less) and the rest to the MoH. "Normally we send confidential forms with our recommendations to the local government or to the MoH for our staff's promotion annually. We send our personnel officer to follow up, with no success. I think the issue is money here. Imagine all the workers employed by the government once they get promotion it comes with money as an incentive. So what (the MoH) do, they give these promotion in a piecemeal."

## Discussion

From the viewpoint of service providers, the main factors identified that caused demotivation among health care workers working at primary health care facilities were workload paired with staff shortages, lack of interprofessional exchange and lack of positive supervision, including transparent career goals. Physical infrastructure and equipment available to staff in the PHC setting did sometimes affect morale – and certainly services – but overall the findings from these focus-group discussions indicate a need for individual staff to feel valued and supported and to develop in their roles.

Data collection is always susceptible to bias. In this survey, particular attention was given to the setting of data collection to maximize the feeling of confidentiality and ease among respondents. During piloting, different mixes of cadres were put together, but no problems were evident between different groups. As far as possible, workers from different facilities and districts were put together to enhance confidentiality.

Similar findings have been reported from Uganda, where professional identity and recognition by both employer and members of the community were found to be important motivating factors for health staff [14]. Further, in Uganda multitasking by unqualified primary health care workers has been cited as a primary factor explaining inefficiencies in the health service [15]. A recent study in northern Tanzania concluded that the lack of adequately trained health workers in rural posts was a major contributor both to the bypassing of primary services by users and to delivery of poor-quality services [16].

Many of the participants in this study felt proud of their ability to cope, but were frustrated that this was not formalized or recognized. While there appeared to be resignation to this, there was also a desire to be trained. Interestingly (and perhaps a reflection of how long some respondents had been acting upwards), while respondents perceived a lack of personnel and lack of personal skill, the principal solution proposed by respondents was not to employ the stipulated number of trained personnel in their facility but rather to give experienced but untrained personnel more formal training. It is also possible that the perceived need for formal training could be confounded by the drive for higher salaries after the training.

In Tanzania, where malaria and HIV/AIDS are priority diseases for control [17], currently there are a number of positive nationwide initiatives to improve detection, treatment and delivery of interventions, such as integrated management of childhood illness (IMCI), discount vouchers for pregnant women to obtain insecticide-treated bed nets and antiretroviral therapy (ARV). However, often the unseen consequence of these initiatives is that there are several competing demands for training health workers how to implement new programmes.

This can mean that health workers are removed from their post for long periods, resulting in an increased burden for those remaining, who are likely to be already weakened by the workload. The challenge posed by scarce human resources in the health system, particularly how to motivate and retain those remaining, has recently been a topic of international debate [18,19]. The participants in this study identified positive supervision and improved feedback from referral hospitals as two achievable measures to improve both moral and quality of health care delivery.

To be trusted by the community was mentioned as a crucial component for motivation of health workers. It was reported in this study that the lack of laboratory diagnosis capacity within the primary health care facilities resulted in health workers' feeling as if they were gambling with patients' health. In malaria-endemic areas, access to microscopic diagnosis is a priority factor in valuing PHC facilities. However, having both microscopes and technicians in place at the same time is not always achievable. Rapid diagnostic tests (RDT) for malaria that can be performed by inexperienced staff may be a way forward in improving this issue [20] although currently the tests are prohibitively expensive.

In our study, health workers complained that their supervision was not systematic – and was not supportive when provided. Several studies have shown that joint problem-solving between supervisors and health workers is essential for quality improvement and job satisfaction [21-23]. However, supervisors themselves are often poorly resourced and may not be trained in effective supervision techniques. This is an important area for development in improving the Tanzanian health service.

## Conclusion

Primary health care facilities are health gatekeepers to the community and it is important that they not be bypassed. To maximize their effectiveness, health workers must be motivated, skilled and supported.

This study has indicated that although financial incentives are important, they are not sufficient to motivate health workers. Supportive supervision, performance appraisal, career development and transparent promotion have been prioritized by PHCF workers for improving the services they deliver in Tanzania.

## Competing interests

The authors(s) declare that they have no competing interests.

## Authors' contributions

RNM contributed to the conception, design, fieldwork, analysis and interpretation of the data and drafting of the manuscript. TCM contributed to the analysis, interpretation and drafting of the manuscript. ICB contributed to the conception, interpretation and drafting of the manuscript.
